# Breastfeeding with HIV: faculty and trainee clinical experience with updated 2023 HHS guidelines

**DOI:** 10.3389/frph.2026.1768530

**Published:** 2026-03-13

**Authors:** Harsimran Bajwa, Daniella Rogerson, Gladys Ramos, Leah Kern

**Affiliations:** 1 Department of Internal Medicine, University of California, San Diego, CA, United States; 2 Department of Obstetrics, Gynecology, and Reproductive Sciences, University of California, San Diego, CA, United States; 3 Department of Pediatrics, University of California, San Diego, CA, United States

**Keywords:** anti-retroviral therapy, breastfeeding, education, HHS guidelines, HIV

## Abstract

**Introduction:**

In 2023, the Department of Health and Human Services (HHS) updated the U.S. prevention of perinatal HIV transmission guidelines to recommend that providers support the decision to breastfeed for people with HIV on antiretroviral therapy with sustained viral suppression.

**Methods:**

We conducted an online survey of University of California, San Diego (UCSD) attendings, fellows and residents from Obstetrics, Gynecology, and Reproductive Sciences (OB/GYN&RS), Pediatrics, and Infectious Disease (ID) departments. Likert scales assessed respondent familiarity with, education on, and comfort with implementing the updated breastfeeding with HIV (BFHIV) guidelines. Kruskal Wallis tests were used to examine differences in familiarity, education, and comfort between respondent characteristics. A *p*-value < 0.05 was considered statistically significant.

**Results:**

Of 51 surveys obtained, 25% of respondents were OB/GYN&RS, 65% were Pediatrics, and 10% adult ID physicians. 50% of respondents reported experience caring for patients with HIV and 65% were familiar with BFHIV guidelines. Prior clinical experience working with a person with HIV who chose to breastfeed was significantly associated with increased familiarity (*p* 0.01) with the guidelines and comfort with implementation (*p* 0.02). There were no differences in familiarity or comfort with implementation based on department or level of training. OB/GYN&RS physicians were significantly less likely to have received education on the guidelines (*p* 0.01).

**Discussion:**

While most UCSD physicians were aware of the updated HHS guidelines, our results demonstrate that as national guidelines change, increased dissemination and clinical education is necessary. Future directions include targeted education for OB/GYN&RS physicians, and further trainee clinical exposure working with people with HIV.

## Introduction

Breastfeeding with HIV (BFHIV) is a complex medical issue. Historically in the United States, prevention of perinatal HIV transmission guidelines recommended strict avoidance of breastfeeding as it the only way to eliminate the risk of postnatal HIV transmission through breastfeeding (1). This contrasts with the World Health Organization (WHO) recommendations for exclusive BFHIV in countries where the risk of infant death from malnutrition, diarrhea, and pneumonia is greater than the risk of infants acquiring HIV through breastfeeding (2).

With maternal antiretroviral therapy (ART) use throughout pregnancy, maternal viral suppression and neonatal post-exposure prophylaxis (PEP) use, research indicates that the risk of HIV transmission through breastfeeding is low <1% but not zero (3–6). In January 2023, the Department of Health and Human Services (HHS) Panel on Treatment of HIV During Pregnancy and Prevention of Perinatal Transmission updated their guidelines regarding BFHIV, recommending: “Individuals with HIV who are on anti-retroviral therapy (ART) with a sustained undetectable viral load and who choose to breastfeed should be supported in this decision” (7).

U.S. medical professional societies have published clinical guidelines consistent with the HHS Perinatal HIV Guidelines, including the American Academy of Pediatrics (AAP) (8) and the HIV Medicine Association of the Infectious Diseases Society of America (IDSA) (9). However, as of October 2025, no American obstetrics, gynecology and reproductive sciences (OB/GYN&RS) professional association has published guidelines on BFHIV.

There is a dearth of literature evaluating provider attitudes on BFHIV since the publication of the updated HHS, AAP, and IDSA guidelines. A survey-based study conducted prior to the updated HHS guidelines found that breastfeeding and HIV medicine providers involved in BFHIV counseling struggled with “the tension between responding to parents’ choices, while simultaneously protecting infants from risk of infection…” (10). A survey conducted after the updated HHS guidelines found that, among Pediatric Infectious Disease specialists and Neonatologists practicing in the United States, physicians with fewer years of experience were less likely to offer breastfeeding support for individuals living with HIV (11). A small qualitative study conducted in 2024 found that while providers welcomed the updated guidelines, the “nonzero transmission risk induced anxiety … for providers” and there was inconsistent provider awareness of the updated guidelines, including across experience levels (12).

The objective of our study was to characterize physician knowledge of the updated guidelines and physician comfort with counseling patients BFHIV at our academic training institution. In addition, we sought to analyze how knowledge and attitudes may vary by level of training or specialty (OB/GYN&RS vs. Pediatrics vs. Infectious Diseases).

## Methods

We conducted an online survey of University of California San Diego (UCSD) attending, fellow and resident physicians from the Departments of Obstetrics Gynecology and Reproductive Sciences (OB/GYN&RS), Pediatrics, and Adult Infectious Diseases to assess physician knowledge and education regarding the HHS guidelines, and their comfort with caring for patients BFHIV. The online survey was distributed to an estimated 200 eligible participants. Specifically, the 200 eligible participants were recruited using email with an online link to the survey sent by Department representatives, including Division chairs and Residency program leadership. Additionally, QR codes with links to the survey were physically and electronically disseminated at in-person Departmental or trainee meetings. Survey responses were obtained from October through December 2024.

Participation was voluntary, and recruitment materials explained that participation would not impact participant academic position. Consent was provided by completing the survey. Survey responses could not be linked back to participants. No protected health information was accessed for this study. The study was approved by the UCSD Institutional Review Board.

The survey included 16 questions, including demographic data (age, gender, race/ethnicity, level of training, field of training). The survey used 5-point Likert scales to assess the degree to which respondents were familiar with and received education regarding national and institutional recommendations, and the degree to which respondents are comfortable implementing and providing counseling/education based on the guidelines. (Supplementary 1 Questionnaire). Lastly, an optional free-text question elicited respondent views towards caring for and counseling patients living with HIV who choose to breastfeed.

Quantitative analyses included descriptive statistics of demographic data. Kruskal Wallis nonparametric testing was used to assess for statistically significant differences in Likert scores for respondent familiarity with the updated HHS guidelines, respondent education regarding the updated HHS guidelines, and respondent comfort with counseling patients BFHIV by variables including department, level of training, and prior experience with BFHIV. Qualitative analyses were performed by analyzing free-text responses for common themes by two study investigators. R Project for Statistical Computing was used for all analyses. A *p*-value <0.05 was considered statistically significant.

## Results

51 surveys were obtained, an estimated 25% response rate of approximately 200 eligible participants. Of 51 respondents, 33 (64.7%) were from the Department of Pediatrics, 13 (25.5%) were from the Department of OB/GYN&RS and 5 (9.8%) were from the Division of Infectious Diseases and Global Public Health. Thirty-one (62%) respondents were attendings, 4 (8%) were fellows, and 15 (30%) were residents. Over 50% identified as female and white. Exactly 50% (*N* = 25) respondents answered that they had prior clinical experience working with or counseling people living with HIV who chose to breastfeed. Demographic data are presented in Table 1.

**Table 1:** Respondent characteristics.

Variable	N (%)
Departmental affiliation	*N* = 51 survey responses
OB/GYN&RS—Generalist or Hospitalist	10 (19.61%)
OB/GYN&RS—Maternal Fetal Medicine	3 (5.9%)
Pediatrics—General Pediatrics	19 (37.3%)
Pediatrics—Newborn Medicine	6 (11.8%)
Pediatrics—NICU	4 (7.8%)
Pediatrics—General Infectious Diseases	4 (7.8%)
Internal Medicine—General Infectious Diseases	2 (3.9%)
Internal Medicine—HIV Medicine	3 (5.9%)
Level of training	*N* = 50 survey responses
Attending	31 (62%)
Fellow	4 (8%)
Resident	15 (30%)
Years in practice	*N* = 51 survey responses
0–5	22 (43.1%)
5–10	8 (15.7%)
10–15	7 (13.7%)
15–20	5 (9.8%)
20+	9 (17.6%)
Gender identity	*N* = 51 survey responses
Female	39 (76.5%)
Male	10 (19.6%)
Decline to answer	2 (3.9%)
Race/ethnicity	*N* = 51 survey responses
Asian	11 (21.6%)
Black or African American	1 (2%)
Hispanic or Latino	6 (11.8%)
White	29 (56.9%)
Decline to answer	4 (7.8%)

When assessing respondent prior experience counseling people living with HIV regarding their potential to breastfeed, a large portion of respondents reported never having performed this counseling (*N* = 15, 31.3%), and most respondents reported only performing this counseling once or twice ever (*N* = 23, 47.9%).

Respondent responses about familiarity with the updated HHS guidelines, education about the guidelines, comfort implementing the guidelines, and comfort providing BFHIV counseling are summarized in Table 2. Overall, most respondents were familiar with the HHS guidelines. There were no statistically significant differences in familiarity with the guidelines across departmental affiliations or level of training. However, respondents with prior experience caring for a person living with HIV who chose to breastfeed reported more familiarity with the guidelines (*p*-value 0.01).

**Table 2:** Respondent familiarity, education, comfort with implementation and comfort with counseling likert scales.

Likert scale variable**	Familiarity with HHS guidelines	Education about HHS guidelines	Comfort with implementation	Comfort with counseling
	Median (*p*-value)*	Median (*p*-value)*	Median (*p*-value)*	Median (*p*-value)*
Department				
OB/GYN&RS	4	**2**	3	3
Pediatrics	5	**3**	4	4
Internal medicine	4 (0.78)	**4 (0.01)**	4 (0.25)	4 (0.60)
Level of training				
Attending	5	3	4	4
Fellow	4.5	2	3	3.5
Resident	4 (0.56)	3 (0.90)	3 (0.08)	3 (0.17)
Gender identity				
Female	4.5	3	4	4
Male	4.5	3	3.5	4
Decline to answer	3 (0.61)	1 (0.08)	2.5 (0.49)	1 (0.24)
Race/ethnicity				
Asian	5	4	4	3
Black	2	2	2	2
Hispanic or Latino	5	3	4	4
White	4	3	4	4
Decline to answer	4 (0.50)	1 (0.60)	4 (0.62)	4 (0.29)
Previously cared for BFHIV				
Yes	**5**	3	**4**	**4**
No	**4 (0.01)**	2 (0.09)	**3 (0.02)**	**3 (0.02)**

* *p*-value for Kruskall Wallis.

** Likert scale.

1, no familiarity/education/comfort. 2, little familiarity/education/comfort. 3, some familiarity/education/comfort. 4, moderate familiarity/education/comfort. 5-significant familiarity/education/comfort.

Bold values are statistically significant *p*-values (< 0.05).

In terms of respondent education about the new HHS guidelines and BFHIV, 21 out of 48 (43.75%) physicians responded that they had received little or no education (Likert scores of 1 or 2). Physicians from the Department of OB/GYN&RS were more likely to report lower Likert scores compared to physicians from the Department of Pediatrics or Internal Medicine-Infectious Diseases (*p* = 0.01) (Table 2). There were no differences in education across level of training or respondent prior experience working with a PLHIV who chose to breastfeed.

When asked about comfort implementing the guidelines into clinical practice, 28 out of 49 (57.1%) respondents shared that they felt comfortable (Likert scores of 4 or 5). When asked about comfort counseling or providing education to people living with HIV who choose to breastfeed, a similar percentage felt comfortable (*N* = 29 out of 50 respondents, 58%). There was no statistically significant difference in comfort with implementation into clinical practice or comfort with counseling/providing education by respondent departmental affiliation, level of training, or race/ethnicity. Respondents with prior experience working with a person living with HIV who chose to breastfeed were significantly more likely to report greater comfort with implementation into clinical practice (*p*-value 0.02) and comfort providing counseling/education (*p*-value 0.02) (Table 2).

Of 25 free-text responses to the question “What are your views, as a clinician, towards counseling people living with HIV regarding breastfeeding?” were obtained. Responses generally fit into one of three broad themes: (1) hesitancy towards counseling regarding BFHIV (2) desire for further education/exposure on BFHIV counseling and (3) supportive of BFHIV counseling (Table 3).

**Table 3:** Example of respondent views on counseling BFHIV.

Hesitant	Desire for education/exposure on BFHIV	Supportive
I feel the data supports the new recommendation for breastfeeding in this select low risk population of HIV patients. Admittedly the change still makes me nervous as we have seen the risks prior to ART and have had the long-standing recommendation against. I would counsel patients about their choices and of course the tremendous benefits of breastfeeding overall.	I think the new guidelines make sense, I just haven't received specific education on them. And I don't know if the rest of the team (pediatricians, lactation consultants, etc) are also aware and on the same page.	Happy to support a PLHIV to breastfeed if that is their choice given existing inequities regarding formula.

## Discussion

In our study of residents, fellows, and attendings in Pediatrics, OB/GYN&RS, and Infectious Diseases, we found that most respondents were familiar with the updated HHS guidelines that individuals with HIV who are on ART with a sustained undetectable viral load who choose to breastfeed should be supported in this decision. However, most respondents had received little to no education on the guidelines, with OB/GYN&RS physicians more likely to report not receiving education.

Most respondents felt comfortable implementing the guidelines into their future clinical practice and providing counseling and education regarding BFHIV, and there were no differences in comfort based on respondent characteristics including departmental affiliation, level of training, gender identity or race/ethnicity. However, respondents with prior experience caring for patients living with HIV who chose to breastfeed were more likely to report feeling comfortable with implementation of the guidelines, with counseling and providing education to BFHIV.

Our findings represent an updated snapshot of physician attitudes regarding BFHIV after the publication of the updated HHS and professional guidelines. In comparison to the national provider survey carried out prior to the publication of the new HHS guidelines (10), our study similarly found limited prior experience in providing counseling and education regarding BFHIV. However, our study found significantly less physician hesitancy or discomfort. One potential explanation for this is that prior to the guideline changes, physicians may have been forced to balance patient autonomy, a changing clinical practice landscape that was shifting towards support for BFHIV, and following institutional and/or federal guidelines. In comparison to the survey of neonatologists and Pediatric Infectious Diseases specialists conducted after the publication of the updated guidelines (11), our study did not find significant differences in physician comfort based on years of clinical experience, albeit our sample size was smaller.

Our study has numerous strengths. The study included multiple clinical specialties representing a broad range of specialties and levels of training. Our study also had a higher survey response rate (25% of all eligible physicians) compared to other studies where the response rate was 0.8% (10) and 10% (11). There was an even split between physicians who had prior experience working with BFHIV and those who did not.

Limitations of our study include a small sample size at a single academic institution. For this reason, our results may not be generalizable to physicians in different practice settings or geographical areas. Additionally, even though survey response rate was relatively high compared to other studies, it represented only a quarter of physicians potentially eligible to participate. Additionally, there may have been some selection bias as many respondents had less than 5 years of experience. This may be because early career physicians are more likely to respond to online surveys and/or attend departmental meetings. Finally, it is important to acknowledge that our study was limited to physicians. HIV care teams are multidisciplinary and may include advanced practice providers, pharmacists, social workers, nurses and lactation specialists, and this study is limited in its focus on physicians.

Future implications of our study include increasing education regarding the updated guidelines, specifically among OB/GYN&RS physicians. Nationally, OB/GYN&RS may benefit from an endorsement of the HHS guidelines from a national professional OB/GYN&RS organization. In addition, given the strong association between experience working with a person living with HIV who chose to breastfeed and physician familiarity with the guidelines and comfort with implementing them, increasing clinical exposure to BFHIV (i.e., through rotations at sites that serve patients living with HIV) may lead to increased comfort and more positive views towards counseling among physicians.

## Data Availability

The raw data supporting the conclusions of this article will be made available by the authors, without undue reservation.
