# Supplementary material for: Breastfeeding with HIV: faculty and trainee clinical experience with updated 2023 HHS guidelines
Source: Front Reprod Health. 2026 Mar 13;8:1768530. doi: 10.3389/frph.2026.1768530 (PMC13021615; doi:10.3389/frph.2026.1768530)
Supplement: Supplementary file 1 [file Datasheet1.pdf]

UNIVERSITY OF CALIFORNIA, SAN DIEGO
EXEMPT INFORMATION SHEET

Note: In this information sheet the word "you" refers to the person being considered for enrollment in the study described. This may be you as the reader of this document, a person for whom you are serving as the Legally Authorized Representative (LAR) or surrogate, or your child.

You are being invited to participate in a research study titled Breastfeeding with HIV: Faculty and Trainee Clinical Experience with Updated 2023 HHS Guidelines. This study is being conducted by Dr. Leah Kern, Dr. Gladys Ramos, and Dr. Amutha Rajagopal from UC San Diego. You were selected to participate in this study because you are a UCSD faculty member, fellow, or resident who may care for people living with HIV who may choose to breastfeed.

The purpose of this research study is to assess UCSD faculty and trainee knowledge of and comfort with the updated US Department of HHS guidelines on Breastfeeding with HIV. Your participation in this research should last approximately 20 minutes. If you agree to take part in this study, you will be asked to complete the following survey.

Your participation in this study is completely voluntary and you can withdraw at any time. Choosing not to participate or withdrawing will result in no penalty or loss of benefits to which you are entitled. You are free to skip any question that you choose.

If you have questions about this project or if you have a research-related problem, you may contact the researcher(s), Dr. Leah Kern at lkern@health.ucsd.edu If you have any questions concerning your rights as a research subject, you may contact the UC San Diego Office of IRB Administration at irb@ucsd.edu or 858-246-4777.

By participating in this research, you are indicating that you are at least 18 years old, have read this information sheet, and agree to participate in this research study. Please keep this information sheet for your records.

Departmental Affiliation
Categorical response
Ob/Gyn – Generalist or Hospitalist
Ob/Gyn – Maternal Fetal Medicine
Pediatrics – General Pediatrics
Pediatrics – Newborn Medicine
Pediatrics -NICU
Pediatrics – Infectious Diseases
Internal Medicine – General Infectious Diseases
Internal Medicine – HIV Medicine/Owen Clinic
Decline to answer

Level of training
Categorical response
Attending
Fellow
Resident

How many years have you been in practice?
Categorical response
0-5
5-10
10-15
15-20
20+

How do you identify your gender?
Categorical response
Female
Male
Nonbinary
Decline to answer

How do you identify?
Categorical response
American Indian or Alaska Native
Asian
Black or African American
Hispanic or Latino
Native Hawaiian or Other Pacific Islander
White
Decline to answer

On a scale of 1-5, 1 being not familiar at all and 5 being very familiar, how familiar are you with the 2023 Department of HHS updated guidelines for breastfeeding among people living with HIV (PLHIV)?
Scale

On a scale of 1-5, 1 being not familiar at all and 5 being very familiar, how familiar are you with the 2023 US Guideline recommendation that people living with HIV (PLHIV) who are on ART with a sustained undetectable viral load and who choose to breastfeed should be supported in this decision?
Scale

On a scale of 1-5, 1 being no education and 5 being significant education, how much education have you received about the Guidelines for breastfeeding among PLHIV?
Scale

For those who received education, how did you receive education on the 2023 US guidelines for breastfeeding among PLHIV? Can select more than one answer.
Categorical response
UCSD-based meeting, conference, educational session, or webinar
Local or national non-UCSD meeting, conference, educational session, or webinar
Other

Have you ever cared for a person living with HIV who breastfed?
Categorical response
Yes
No

Have you counseled people living with HIV about their infant feeding choices (breastfeeding vs formula feeding) in your practice? If so, how often?
Categorical response
At least weekly
At least monthly
At least 5 times in the past year
At least 5 times in the past 5 years
Once or twice ever
Never

Have you counseled a person living with HIV about their potential to breastfeed in your practice? If so, how often?
Categorical response
At least weekly
At least monthly
At least 5 times in the past year
At least 5 times in the past 5 years

Once or twice ever
Never

On a scale of 1-5, 1 being not comfortable at all and 5 being very comfortable, how comfortable are you with implementing the Department of HHS updated guidelines for breastfeeding among PLHIV?
Scale

On a scale of 1-5, 1 being not comfortable at all and 5 being very comfortable, how comfortable are you counseling/providing education to people living with HIV who choose to breastfeed?
Scale

What, if any, gaps do you believe exist that prevent you, as a clinician, from effectively counseling PLHIV regarding breastfeeding? Can select multiple.
Categorical response*
None
Lack of education about counseling PLHIV regarding their potential benefits and risks of breastfeeding
Concern for a lack of data regarding PLHIV and breastfeeding
Lack of time during visits
Lack of awareness of the UCSD Health policy on counseling PLHIV regarding breastfeeding
Discomfort
Ethical concerns
Concern for risk of HIV transmission to infant
Language barriers
Cultural barriers
Need more support from UCSD care team
Comfortable with routine breastfeeding counseling but not sure what to do in more complicated situations such as mastitis, thrush, milk protein allergy, etc
Other (write in text)

What are your views, as a clinician, towards counseling PLHIV regarding breastfeeding?
Free text
